# Treatment of Wounds of the Pelvic Basin

**Published:** 1918-09

**Authors:** 


### UROLOGY AND DERMATOLOGY

---

***Treatment of Wounds of the Pelvic Basin, Especially of the Bladder and Rectum.*** By M. J. Tanton. Report presented at *the Second Session of the Inter-Allied Surgical Conference.*

Tanton discusses the subject in four divisions, viz :

1. Isolated wounds of the pelvic basin.

2. Isolated wounds of the bladder; with or without concomitant lesions of the bony basin.

3. Isolated wounds of the rectum; with or without concomitant wounds of the bony basin.

4. Associated wounds of the bony basin.

*Isolated Wounds of the Bony Basin.* Under this heading is discussed the wounds that do not penetrate the abdomen because the bony lesion is considered of secondary importance to the intestinal lesions. 3719 cases were studied. Of these there were 1659 lesions of the ilium; 311 of the iliac crest; 659 of the sacrum; 24 of the coccyx; 21 of the sacrum and the coccyx; 20 of the wing of the sacrum; 120 of the sacro-iliac region; 202 of the pubic bone; 12 of the pubic symphisis; 47 of the ischio-pubic rami; 241 of the ischium; 18 of the ilium and one ischio-pubic ramus; 52 of the ilium and of the acetabulum; and 321 wounds, the site of which was not precisely known.

2120 of the wounds resulted from shells, 773 from bullets, 136 from grenades and 108 from shrapnel.

The mortality rate was 10.37 o/o; 7.37 o/o died immediately and the secondary mortality was 4 o/o.

*Anatomo-pathological Lesions.* The penetration of the projectile, in the greatest number of cases, is produced through the hip, which explains the enormous predominance of lesions of the iliac wing; sometimes through the perineum, the lesion then involving the pubic bone, the ischio-pubic ramus or ischium; very often through the sacral region. Fracture of the head and neck of the femur is frequently associated. The lesions of the soft parts are variable. Sometimes in extensive fractures of the crest of the ilium the insertions of the large muscles of the abdomen and of the gluteals are destroyed. There also may be lesions of the sciatic trunk, or the sacral plexus.

*Evolution. Complications.* Shock is often severe and hemorrhages frequent because of the injury to the gluteal vessels. Infections develop rapidly on account of the extensive trauma. Gas gangrene finds here some favorable conditions for development. Rupture of the ureter sometimes causes a sub-peritoneal urinary phlegmon. Secondary hemorrhage and septicemia enter largely in the secondary mortality from lesions of the bony pelvis.

*Treatment.* Early intervention reduces the mortality. Wide excision of the contused soft parts, total esquillectomy, wide trephination of the iliac bone to remove the projectile and primitive suture of the soft parts are indicated when expedient. When infection occurs free drainage is demanded. In pelvic cellulitis the resection of the coccyx drains in a complete fashion.

*Wounds of the Bladder.* Associated lesions of the bladder and bony basin were more frequent than uncomplicated vesical injuries. The extra-peritoneal portion of the bladder was injured in 266 cases, while in 68 the peritoneal portion was involved, the latter being often accompanied by concomitant lesions of the small intestines and the colon.

*Treatment.* The author is convinced that every confirmed, or simply suspicioned, vesical lesion imposes an immediate surgical intervention. When the lesion is intra-peritoneal, or supposedly such, a laparatomy should be performed and the bladder and intestines carefully explored. The projectile will have been previously located by the X-ray and removed if it is in the bladder. The contused borders of the vesical perforation should be excised, and sutured with catgut in two places, the intestinal wounds also sutured, if any exist, and the vesical cul-de-sac carefully dried out, the abdomen closed, leaving a drain in the pouch of Douglas for two or three days. He does not consider a retention catheter indispensable, if the bladder is catheterized every three hours for the first four or five days. The mortality rate of bladder wounds is high, 75 o/o of the cases reported, when the secondary deaths are included.

The indications for treatment of extra-peritoneal wounds of the bladder may be summed up as follows : surgical disinfection of the wound of entrance, and primary supra-pubic cystostomy in most cases. Experience has shown that many wounds of the bladder, by bullets or shell splinters, heal spontaneously.

